# First-in-human experience for cardiac arrhythmia mapping using a novel ultra-high–density globe-shaped catheter from the multicenter COSMOS study

**DOI:** 10.1016/j.hroo.2025.11.029

**Published:** 2026-01-08

**Authors:** Ante Anic, Massimo Grimaldi, Tom De Potter, Carlo de Asmundis, Gian Battista Chierchia, Federico Quadrini, Antonio di Monaco, Toni Brešković, Moussa Mansour, Hiroshi Nakagawa, Alexandre Almorad

**Affiliations:** 1 University Clinical Hospital Split, Split, Croatia; 2 Ospedale Generale Regionale F. Muilli, Acquaviva delle Fonti, Italy; 3 Onze Lieve Vrouwziekenhuis Hospital, Aalst, Belgium; 4 Universitair Ziekenhuis Brussel-Vrije Universiteit Brussel, Laarbeeklaan, Brussels, Belgium; 5 Massachusetts General Hospital, Boston, Massachusetts; 6 Cleveland Clinic, Cleveland, Ohio

**Keywords:** Atrial tachycardia, Atrial flutter, Premature ventricular complex, Paroxysmal atrial fibrillation, Persistent atrial fibrillation, Ventricular tachycardia, High resolution, Electroanatomic mapping

## Abstract

**Background:**

A novel ultra-high–resolution mapping system (Clarysense, Biosense Webster, Inc) has been developed that consists of a 100-electrode deflectable bidirectional basket-shaped high-density mapping catheter that incorporates a central TRUEref electrode and is integrated with the CARTO 3 system.

**Objective:**

This study aimed to assess the clinical safety and performance of the novel system for mapping in complex atrial and ventricular arrhythmias.

**Methods:**

This prospective, single-arm, multicenter study included patients undergoing catheter mapping and ablation of atrial and ventricular arrhythmias. Mapping was performed with the study catheter, and participants were ablated per the investigator’s standard of care. The primary effectiveness endpoint was completion of preablation electroanatomic mapping without resorting to a nonstudy catheter. The primary safety endpoint was the incidence of device-related serious adverse events within 7 days. Physician feedback on catheter performance was collected using a 7-point Likert scale.

**Results:**

40 participants (mean age 58.0 ± 15.73 years; 62.5% male; 30 with atrial arrhythmias, 10 with ventricular arrhythmias) underwent mapping with the study catheter. The primary effectiveness endpoint was achieved in all participants; ≥1 area of interest for mappable rhythms was identified in 23 of 30 participants with atrial arrhythmia. One serious adverse event of transient complete atrioventricular block was reported in a participant with persistent atrial fibrillation during preablation mapping, with full recovery. Physician feedback indicated that the device met or exceeded expectations for signal quality; most responders rated highly on bipolar signal quality in atria and noise encountered.

**Conclusion:**

In this first-in-human study, the novel mapping system improved high-density mapping in complex arrhythmias with high-quality signals and map resolution. The catheter exceeded operators’ expectations in signal fidelity.

## Graphical abstract

**Figure:**
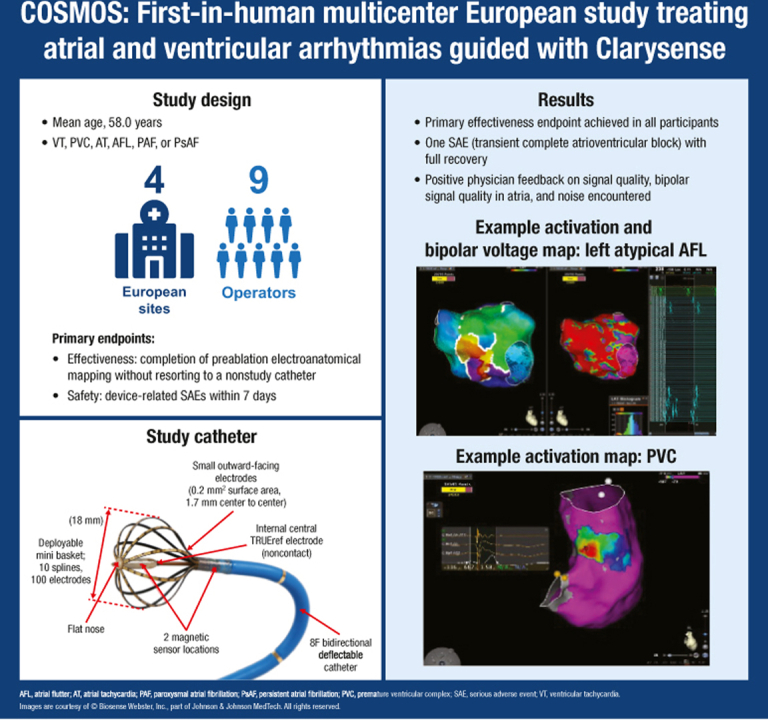

**Key Findings**

▪ Clarysense is a novel ultra-high–resolution mapping system that consists of a 100-electrode deflectable bidirectional basket-shaped high-density mapping catheter with TRUEref electrodes.
▪ In the COSMOS first-in-human study, preablation electroanatomic mapping was achieved using the study catheter alone.
▪ Only 1 device-related serious adverse event was reported.
▪ Physician feedback indicated that the mapping catheter met or exceeded their expectations for deployment, maneuverability, and signal quality, with physicians citing the reduced arrhythmogenicity of the catheter design as a particular benefit relative to other devices.

## Introduction

The advancement in high-density diagnostic catheters and mapping systems has contributed to substantially more efficient procedures and improved ablation outcomes than conventional systems.^1^, ^2^, ^3^, ^4^, ^5^ In the ablation of complex arrhythmias, high-density, high-resolution mapping has enhanced the accurate localization of regions of slow conduction (critical isthmus) or macroreentrant circuits and the earliest activation of focal tachycardias.^6^^,^^7^ In addition, rapid electrogram collection with higher density and improved resolution has given rise to shorter mapping and procedure times. In atrial fibrillation (AF) ablation procedures, high-density mapping has the potential to visualize previously concealed gaps, thereby improving pulmonary vein isolation (PVI) durability and reducing the need for reablation.^4^^,^^8^

However, in practice, current high-density mapping systems are associated with certain limitations. Interference from far-field signals or noise can result in poor signal fidelity. In complex atrial substrates with multiple atrial potential components, unipolar electrograms referenced to the Wilson Central Terminal are often timed to the wrong component owing to large, steep far-field potentials; this can require manual review and corrections of the annotations. Although the use of a close-unipolar reference electrode rather than a conventional unipolar electrogram referenced to the Wilson Central Terminal has been shown to improve mapping accuracy, improvements in signal fidelity have unknown clinical significance.^9^^,^^10^ Other limitations include electrode spacing and sizing, which affect the integrability of ablation and intracardiac echocardiography.

A novel ultra-high–resolution mapping system (Clarysense, Biosense Webster, Inc, part of Johnson & Johnson MedTech, Irvine, CA) has been developed that consists of a mini basket-shaped high-density and high-resolution mapping catheter that includes a central noncontact close-unipolar reference electrode integrated with an electroanatomic mapping (EAM) system, the CARTO 3 system (Biosense Webster, Inc, part of Johnson & Johnson MedTech). In preclinical beating-heart animal studies, the system was shown to rapidly create high-resolution maps that ranged from 924 to 1496 points per minute.^11^^,^^12^ In addition, the use of the central noncontact close-unipolar reference electrode has been shown to reduce far-field signal components, reduce the need for manual annotation corrections, and improve mapping accuracy. Thus far, the usability and safety of the novel system in patients with atrial or ventricular arrhythmias have not been investigated. This first-in-human study aimed to assess the safety and mapping performance of the noncommercially available, globe-shaped catheter with the CARTO 3 system for mapping in the atria and ventricles in patients with a variety of complex arrhythmias.

## Methods

### Study design

The COSMOS study (ClinicalTrials.gov identifier: NCT05373862) was a prospective, single-arm, open-label study conducted by 9 operators at 4 European sites in Belgium, Croatia, and Italy. The primary objective was to assess the safety and performance of the investigational catheter for intracardiac mapping in the atria and ventricles. A secondary objective was to gather physicians’ assessment of deployment, maneuverability, and signal quality acquired with the investigational catheter for mapping in the atria and ventricles.

### Device description

**Figure 1:**
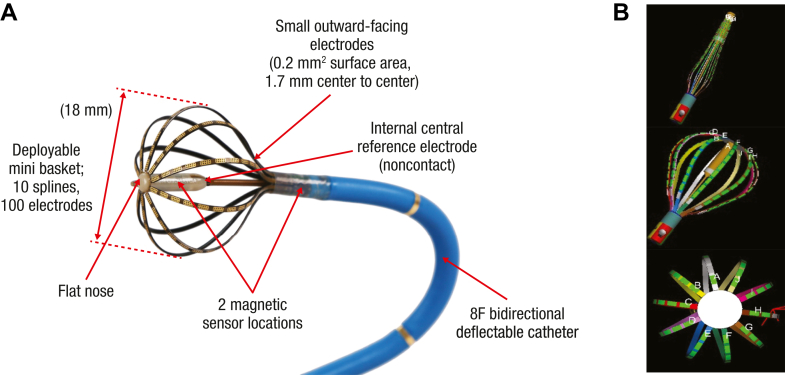
Catheter design and visualization. **A:** Deployable mini basket with 100 electrodes (each 0.2 mm^2^ surface area) and a 1.7 mm spacing over 10 splines with center and proximal magnetic sensors that allows basket visualization and orientation. A central noncontact unipolar reference electrode is in the center of the basket. **B:** Catheter visualized on the electroanatomic mapping system in a closed and open shape. Images are courtesy of © Biosense Webster, Inc, part of Johnson & Johnson MedTech. All rights reserved.

The deflectable bidirectional mapping catheter is designed with 100 electrodes coated over 10 splines to form a basket shape. It has an 8F shaft compatible with an 8.5F sheath for advancement into the heart chamber (Figure 1). The basket has an adaptable size for the diameter, from a minimum of 3 mm to a maximum of 18 mm, and maps can be acquired throughout the range of deployment depending on the need at a particular anatomic location. The basket array consists of 10 nitinol spines and a laminated flexible printed circuit with 10 tiny outward-facing gold electrodes (surface area of 0.5 mm^2^ and 1.7 mm interelectrode spacing, center-to-center) on each spline (total of 100 electrodes), connected distally to a flat-nose tip. The electrodes are flat with an impedance-reducing coating to allow for decreased electrical impedance, improved signal quality, and superior signal-to-noise ratio compared with previous high-density mapping catheters.

Three magnetic sensors, 2 in the distal portion and 1 in the proximal portion, are embedded in the basket array that transmit location and angle information independent of advanced catheter location to the CARTO 3 system, with a system accuracy of <1 mm. Below the splines on the deflectable tip are 2 electrodes that allow visualization of the shaft on the CARTO 3 system. The TRUEref electrode (Biosense Webster, Inc, part of Johnson & Johnson MedTech) is embedded in the center of the globe and can be used as a noncontact central reference electrode for close-unipolar electrograms within the heart chamber, which allows enhanced qualifying and filtering of incoming signals for map annotation. CARTO 3 software version 7 is compatible with reducing the need for manual annotation; an added feature included an automatic outlier detection and binning of points algorithm, termed “Wisdom of the crowd” (WofC). Briefly, this feature removes outlier timing or voltage annotation based on a certain criterion collected within a 1-mm^3^ predefined fast anatomic mapping (FAM) area (voxel).

### Study endpoints

The primary effectiveness endpoint was the completion of protocol-required electroanatomic preablation mapping without resorting to nonstudy catheters. The primary safety endpoint was the incidence of device-related serious adverse events (SAEs) within 7 days after the procedure.

The secondary effectiveness endpoint was the deployment characterization, maneuverability, and signal quality acquired with the investigational catheter in the atria and ventricles based on physicians’ feedback on the postprocedure survey. The survey was collected after each study procedure for each investigational catheter used and included questions with Likert-scale response options for maneuverability and handling, signal collection and quality, pacing, catheter design, workflow, visualization, catheter’s interactions, arrhythmogenicity, design and coverage for confirming PVI, and ability to characterize the tissue. This structured questionnaire was designed to evaluate experienced operator satisfaction with map accuracy/fidelity and a number of investigational catheter features (arrhythmogenicity, design and coverage for confirming PVI, ability to characterize tissue, and ability to identify arrhythmia circuit or source correctly) compared with the operators’ previous experience with the standard of care PENTARAY catheter (Biosense Webster, Inc, part of Johnson & Johnson MedTech), a high-density pentaspline mapping catheter. A score of “4” on the 7-point scale was considered comparable with other devices. The secondary safety endpoint was the incidence of SAEs, excluding investigational catheter-related SAEs within 7 days of the index procedure and the incidence of nonserious adverse events within 7 days of the index procedure related to the investigational catheter.

Additional procedural characteristics were total procedure time, initial mapping duration (time between the first and last mapping point before the first ablation point, as measured on CARTO), areas of interest captured (eg, pulmonary vein triggers, previous PVI lesion gaps, slow conduction scar zone, critical isthmus, etc), and mapping density.

Arrhythmia mapping was assessed as an additional endpoint. An atrial reference was used to determine the cycle length of atrial tachycardias (ATs). The window was adjusted to include points across the entire atrial cycle length or pre–P-wave for atrial flutter (AFL) or focal AT, respectively, and automatic points acquisition (CONFIDENSE) was set to the individual operator’s discretion. A ventricular reference was used to determine the cycle for ventricular tachycardias (VTs) or mapping of premature ventricular complexes (PVCs). The window was adjusted to include points across the entire ventricular cycle length or pre-QRS wave for sustained VTs or PVCs, respectively, and automatic points acquisition (CONFIDENSE) was set to the operator’s discretion.

### Study participants

Eligible participants were scheduled to have a clinically indicated catheter mapping and ablation procedure for VT, PVC, AT, AFL, or paroxysmal AF (PAF) or persistent AF (PsAF); patients having undergone a previous ablation procedure could be included. The exclusion criteria included diagnosis of an arrhythmia that requires epicardial mapping, left ventricular ejection fraction of ≤25% for patients with VT, and left ventricular ejection fraction of ≤40% for patients with atrial arrhythmia.

Preprocedure assessment and data collection included baseline medical, cardiac, arrhythmic, and ablation history; transthoracic echocardiogram; pregnancy test; thrombus screening; collection of any adverse events since enrollment; and subsequent ablation according to institutional standard of care practice.

### Study procedure

The investigational catheter was used solely for all preablation mapping procedures. The catheter was advanced into the cardiac chamber of interest via any commercially available 8.5F sheath, and maps were created with automatic acquisition (CONFIDENSE) of points for each beat gated to the respiratory and cardiac cycle. Preablation mapping included FAM of the entire chambers and areas associated with the targeted arrhythmia. Electroanatomic mapping was performed to determine substrate voltage or tachycardia activation mechanism and local activation timing (LAT); identify conduction channels, gaps, and critical isthmuses; and determine an adequate level of mapping density at the area of interest. The investigational catheter was used with continuous irrigation and an activated clotting time of ≥300 seconds. Heparinized saline (1 unit/mL) was infused through the central lumen of the catheter shaft at a rate of 2 mL/min and emerged at the end of the shaft (proximal end of the basket) to prevent thrombus.

After mapping, the ablation procedure was performed as per the institution’s standard of care. If additional mapping was clinically indicated after ablation, the investigational catheter was used. Phrenic nerve pacing was frequently performed during catheter ablation to assess any nerve damage by the ablation catheter. The ability of the study catheter to conduct phrenic nerve pacing or pacing capture was examined. The follow-up period was 7 days with a telephone call or an in-person clinic visit to assess for adverse events.

### Statistical analysis

There was no formal hypothesis testing on outcomes in this study. All data were summarized using descriptive analyses, with confidence intervals using a 2-sided 95% confidence level unless otherwise stated. A score of 4 on a 7-point Likert scale indicated that the catheter met expectations or was comparable with other devices. Scores of ≥4 were considered as successful, with 1 = poor and 7 = excellent.

This study was approved by the respective ethics committees, competent authorities, and regulatory agencies for each study site. Each participant gave written informed consent before study treatment in accordance with the principles of informed consent in the latest version of the Declaration of Helsinki and the requirements and procedures described in the study protocol.

## Results

### Study participants

Patients were enrolled between July 2022 and February 2023; a total 40 patients completed the study procedures. The mean age of participants was 58.0 years and 25 (62.5%) were male (Table 1). Most patients (38 of 40; 95.0%) were without structural heart disease. Approximately two-thirds had no heart failure (27 of 40; 67.5%), whereas 9 patients (22.5%) had New York Heart Association class I and 2 participants (5.0%) had New York Heart Association class II heart failure. Of the 30 participants with atrial arrhythmia (7 with AFL, 2 with AT, 7 with PsAF, and 14 with PAF), 16 patients (53.3%) had a history of ≥1 ablation procedure, which were performed for the treatment of AF (n = 15), typical AFL (n = 5), AT (n = 2), or atypical AFL (n = 2). These previous ablation procedures were performed using radiofrequency (14 procedures), cryoablation (1 procedure), and other ablation technologies (2 procedures). Of the 10 patients with ventricular arrhythmia (1 with VT and 9 with PVC), 2 patients had previous ablations for the treatment of AF (1 patient) and PVC (1 patient), both with radiofrequency catheters (Table 2).

**Table 1:** Baseline demographic characteristics and comorbidities of enrolled participants

Characteristic	Atrial arrhythmia (n = 30)	Ventricular arrhythmia (n = 10)	All arrhythmias (N = 40)
Age, y, mean (SD)	63.2 (11.87)	42.5 (16.17)	58.0 (15.73)
Sex, male, n (%)	19 (63.3)	6 (60.0)	25 (62.5)
Comorbidities, n (%)			
Hypertension	15 (50.0)	1 (10.0)	16 (40.0)
Type 2 diabetes	5 (16.7)	0	5 (12.5)
Coronary artery disease	4 (13.3)	0	4 (10.0)
Thromboembolic events/TIA	1 (3.3)	0	1 (2.5)
Congestive heart failure	0	1 (10.0)	1 (2.5)
Heart failure with NYHA class, n (%)			
Class I	8 (26.7)	1 (10.0)	9 (22.5)
Class II	2 (6.7)	0	2 (5.0)
LVEF, %, mean (SD), [min, max]	57.8 (7.24) [45, 76]	62.9 (7.87) [50, 75]	59.1 (7.63) [45, 76]
LA diameter, mm, mean (SD) [min, max]	43.7 (7.41) [30, 55]	36.1 (6.25) [26, 45]	41.9 (7.79) [26, 55]

LA = left atrial; LVEF = left ventricular ejection fraction; max = maximum; min = minimum; NYHA = New York Heart Association; SD = standard deviation; TIA = transient ischemic attack.

**Table 2:** History of atrial and ventricular arrhythmias and ablation procedures

Variable	Atrial arrhythmia				Ventricular arrhythmia		
	AT/AFL (n = 9)	PsAF (n = 7)	PAF (n = 14)	Total atrial arrhythmias (N = 30)	VT (n = 1)	PVC (n = 9)	Total ventricular arrhythmias (N = 10)
Duration of primary arrhythmia since the first onset (mo)							
n	9	7	14	30	1	9	10
Mean (SD)	24.07 (19.318)	41.97 (43.375)	105.06 (109.629)	66.04 (85.448)	20.00 (–)	11.44 (10.101)	12.30 (9.900)
Median	28.00	24.00	72.00	33.50	20.00	12.00	12.00
Min, max	0.1, 50.6	6.0, 132.0	7.0, 360.0	0.1, 360.0	20.0, 20.0	2.0, 36.0	2.0, 36.0
Average duration of arrhythmia episodes (h)							
n	9	7	14	30	1	9	10
Mean (SD)	118.78 (229.499)	624.04 (775.795)	14.31 (19.485)	187.92 (448.517)	0 (–)	41.89 (74.844)	37.70 (71.796)
Median	24.00	240.00	5.50	24.00	0	24.00	24.00
Min, max	2.0, 720.0	0.3, 1920.0	0, 72.0	0, 1920.0	0, 0	3.0, 240.0	0, 240.0
Previous catheter ablation procedures for arrhythmia							
Yes	6 (66.7)	2 (28.6)	8 (57.1)	16 (53.3)	0	2 (22.2)	2 (20.0)
No	3 (33.3)	5 (71.4)	6 (42.9)	14 (46.7)	1 (100)	7 (77.8)	8 (80.0)
Number of ablations							
n	6	2	8	16	0	2	2
Mean (SD)	1.7 (1.21)	1.0 (0)	2.0 (1.41)	1.8 (1.24)	–	1.5 (0.71)	1.5 (0.71)
Median	1.0	1.0	1.5	1.0	–	1.5	1.5
Min, max	1, 4	1, 1	1, 5	1, 5	–	1, 2	1, 2
All arrhythmias ablated in previous procedures∗							
AF	5 (83.3)	2 (100)	8 (100)	15 (93.8)	0	1 (50.0)	1 (50.0)
Typical AFL	3 (50.0)	1 (50.0)	1 (12.5)	5 (31.3)	0	0	0
Atypical AFL	2 (33.3)	0	0	2 (12.5)	0	0	0
AT	1 (16.7)	0	1 (12.5)	2 (12.5)	0	0	0
AVNRT	0	0	0	0	0	0	0
VT	0	0	0	0	0	0	0
PVC	0	0	0	0	0	1 (50.0)	1 (50.0)
Other	0	0	0	0	0	0	0
Technology used in the most recent ablation procedure∗							
Radiofrequency	5 (83.3)	2 (100)	7 (87.5)	14 (87.5)	0	2 (100)	2 (100)
Cryoablation	0	0	1 (12.5)	1 (6.3)	0	0	0
Other	2 (33.3)	0	0	2 (12.5)	0	0	0

AF = atrial fibrillation; AFL = atrial flutter; AT = atrial tachycardia; AVNRT = atrioventricular reciprocating tachycardia; max = maximum; min = minimum; PAF = paroxysmal atrial fibrillation; PsAF = persistent atrial fibrillation; PVC = premature ventricular contraction; SD = standard deviation; VT = ventricular tachycardia.

∗ Participants are counted in multiple categories; therefore, percentages may total more than 100%.

### Primary effectiveness endpoint

All patients (40 of 40; 100%) completed the protocol-required preablation mapping using the investigational catheter. The entire chambers of interest targeted for arrhythmia mapping were completed with FAM. No other nonstudy catheter was used for any preablation mapping.

### Procedural characteristics

For atrial procedures, the median total procedural duration was 127.0 minutes (140.0 minutes for patients with AT/AFL, 125.0 minutes for patients with PsAF, and 111.0 minutes for patients with PAF), whereas the median total preablation mapping time was 22.2 minutes (22.4 minutes for patients with AT/AFL, 22.0 minutes for patients with PsAF, and 21.8 minutes for patients with PAF). For ventricular procedures, the median total procedural duration was 110.0 minutes (219.0 minutes for the 1 patient with VT and 110.0 minutes for the patients with PVC), whereas the median total preablation mapping time was 14.8 minutes (5.4 minutes for the patient with VT and 16.8 minutes for patients with PVC) (Table 3).

**Table 3:** Procedural characteristics

Category	Atrial arrhythmia				Ventricular arrhythmia		
	AT (n = 9)	PsAF (n = 7)	PAF (n = 14)	Total atrial arrhythmias (N = 30)	VT (n = 1)	PVC (n = 9)	Total ventricular arrhythmias (N = 10)
Total duration of procedure (min)∗							
n	9	7	14	30	1	9	10
Mean (SD)	141.0 (27.76)	138.0 (52.40)	122.0 (44.09)	131.4 (41.64)	219.0 (–)	105.3 (27.13)	116.7 (44.12)
Median	140.0	125.0	111.0	127.0	219.0	110.0	110.0
Min, max	102, 190	75, 217	68, 210	68, 217	219, 219	69, 156	69, 219
Duration of preablation mapping (min)†							
n	9	7	14	30	1	9	10
Mean (SD)	23.8 (11.62)	21.6 (5.58)	26.8 (14.63)	24.7 (12.02)	5.4 (–)	17.9 (9.83)	16.7 (10.07)
Median	22.4	22.0	21.8	22.2	5.4	16.8	14.8
Min, max	3, 41	11, 30	2, 51	2, 51	5, 5	8, 38	5, 38

AT = atrial tachycardia; max = maximum; min = minimum; PAF = paroxysmal atrial fibrillation; PsAF = persistent atrial fibrillation; PVC = premature ventricular contraction; SD = standard deviation; VT = ventricular tachycardia.

∗ Duration of total procedure is defined as the time between the start of the procedure and end of the procedure.

† Duration of preablation procedure is defined as the time between the first and last mapping point before the first ablation point as measured on CARTO.

FAMs were created in all participants, with voltage maps created in all 30 atrial procedures and 8 of 10 ventricular procedures. In addition, LAT maps were created in 25 of 30 atrial procedures and 7 of 10 ventricular procedures. The investigational catheter was used for phrenic nerve pacing in 5 participants, with local pacing capture demonstrated in all of these procedures. Phrenic nerve stimulation was not performed in any procedure. In addition, 30 of 40 patients (75.0%) had post–standard of care mapping performed.

### Safety

One SAE related to the investigational catheter was reported (1 of 40; 2.5%), which was an event of transient complete atrioventricular block during preablation mapping procedure using the investigational catheter in a participant with PsAF. The patient returned to normal rhythm intraprocedurally and was only paced for a short duration during the procedure. The event was considered moderate in severity, and the participant fully recovered without sequalae and was discharged from the hospital the day after the ablation procedure.

Three procedure-related SAEs (3 of 40; 7.5%) unrelated to the investigational catheter were reported, which included 1 atrioventricular block, 1 cardiac tamponade, and 1 pericardial effusion, all in participants with AT. The participant with atrioventricular block required pacemaker implantation and was discharged 7 days after the procedure; the event was considered possibly related to the ablation procedure. The 2 participants with cardiac tamponade and pericardial effusion required pericardial drainage and were discharged from the hospital after the ablation procedure at 3 and 2 days, respectively. The investigation report considered both cardiac tamponade and pericardial effusion to be SAEs, not related to the investigational device, and with an anticipated and causal relationship to the procedure.

One event of transient right bundle branch block during preablation mapping in a ventricular procedure was deemed to be a nonserious adverse event related to the investigational catheter. The event was classified as nonserious owing to no medical or surgical intervention taken and was resolved spontaneously without medical intervention.

No embolic events were reported. At the end of the procedure, catheters were inspected, and no visible thrombus, device malfunctions, or cardiac structure entanglement was reported.

### Operator feedback

**Figure 2:**
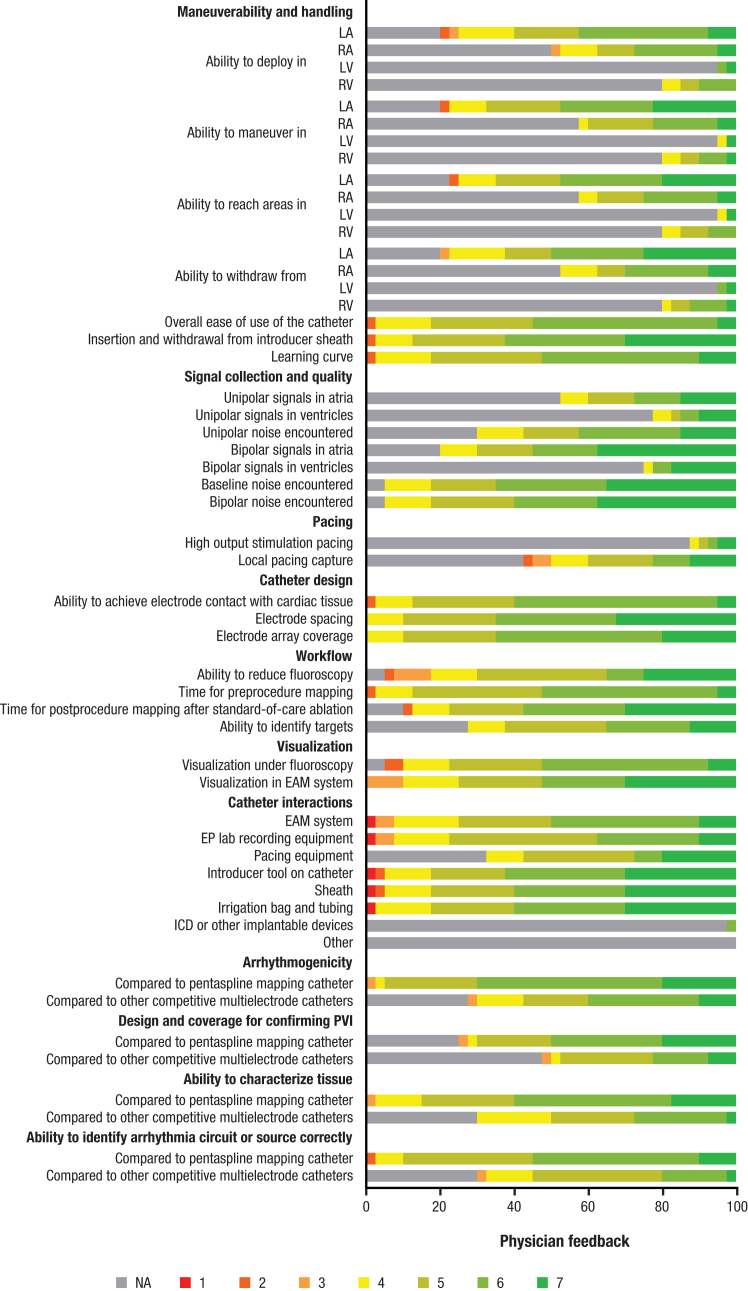
Operator feedback on the investigational catheter. Using a 7-point Likert-scale survey (1 = poor and 7 = excellent), the feedback on maneuverability and handling, pacing, unipolar and/or bipolar signal quality, workflow, visualization, and catheter interactions was assessed by the operators. The investigational catheter features were compared with the operators’ previous experience using the PENTARAY catheter. Images are courtesy of © Biosense Webster, Inc, part of Johnson & Johnson MedTech. All rights reserved. EAM = electroanatomic mapping; EP = electrophysiology; ICD = implantable cardioverter-defibrillator; LA = left atrium; LV = left ventricle; NA = not assessed; PVI = pulmonary vein isolation; RA = right atrium; RV = right ventricle.

The 7-point Likert scale indicated that the investigational catheter met or exceeded physicians’ expectations in terms of deployment, maneuverability, and signal quality acquired for mapping in the atria and ventricles (Figure 2). The investigational catheter was scored mostly as “meeting expectations” or “better than expectations” in its deployment and maneuverability in the atria and ventricles. Regarding signal quality, unipolar and bipolar signals acquired with the investigational catheters were all rated as meeting or better than expectations. Overall, the catheter was considered equal to or an improvement over a pentaspline mapping catheter in its arrhythmogenicity and ability to characterize tissue and confirm PVI, as applicable. An operator learning-curve effect could not be fully excluded.

### Arrhythmia mapping

In all 30 atrial arrhythmia procedures, FAM and voltage maps were completed. LAT mapping was performed in 83.3% of participants (25 of 30), including all 9 focal AT/AFL procedures (100%), 11 of 14 PAF procedures (78.6%), and 5 of 7 PsAF procedures (71.4%). FAMs were also created in 100% of participants with VT (10 of 10). Voltage mapping was performed in 80.0% of the participants (8 of 10) and LAT in 70.0% of participants (7 of 10).

**Figure 3:**
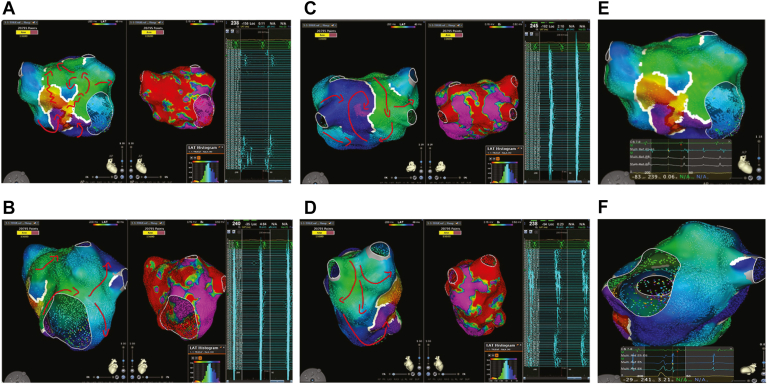
Atypical left atrial flutter mapping. **A–D:** Complex macroreentrant circuits of left atrial atypical flutter timing (LAT) and voltage (bipolar) maps. The entire tachycardia cycle length (∼240 ms) was mapped, and the circuit was identified. Areas of low voltage or scar were also defined with adjusted voltage cutoff (<0.1 mV in *red* and >0.5 mV in *purple*). On the right side, the intracardiac electrocardiogram recordings were observed by the study catheter. **E and F:** Filtering out far-field ventricular signals during atrial tachycardia mapping and around lines of block, reducing incorrect annotations. Images are courtesy of © Biosense Webster, Inc, part of Johnson & Johnson MedTech. All rights reserved. LAT = local activation time.

High-density, high-resolution LAT mapping helped identify and visualize atypical tachycardia mechanisms. The close mapping reference electrode on the catheter allowed operators to acquire points with a clearer signal quality; therefore, signals were annotated to the correct near-field component instead of the far-field. In addition, the WofC algorithm effectively and automatically reduced outlier LAT annotation based on the density of data collected within a 1-mm^3^ voxel (Figure 3).

**Figure 4:**
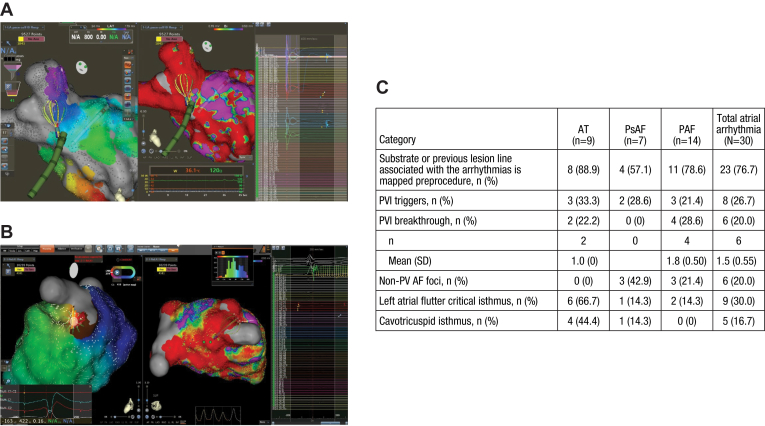
PV breakthrough and focal AT mapping. **A:** PVI breakthrough was identified and mapped at the anterior right PV using the investigational catheter. **B:** The earliest QS pre–P-wave focal AT site was mapped and identified at the anterior left carina area. **C:** Table summarizing the areas of interest identified by the investigational catheter in participants with AT to include PVI triggers, PVI breakthroughs, non-PV AF foci, left atrial critical isthmus, and cavotricuspid isthmus. Images are courtesy of © Biosense Webster, Inc, part of Johnson & Johnson MedTech. All rights reserved. AF = atrial fibrillation; AT = atrial tachycardia; PAF = paroxysmal atrial fibrillation; PsAF = persistent atrial fibrillation; PV = pulmonary vein; PVI = pulmonary vein isolation; SD = standard deviation.

Acquired points were automatically annotated to the sharpest greatest negative deflection (−dV/dt) and delineated the earliest pre–P-wave intracardiac atrial signal with a QS complex and a sharp downstroke for focal ATs. Although the earliest PVI breakthrough site was targeted to achieve vein isolation, 23 of 30 participants with AT (76.7%) had 1 or more areas of interest, such as pulmonary vein and non–pulmonary vein triggers, PVI breakthrough, left AFL critical isthmus, or cavotricuspid isthmus, identified by the investigational catheter (Figure 4A–4C).

**Figure 5:**
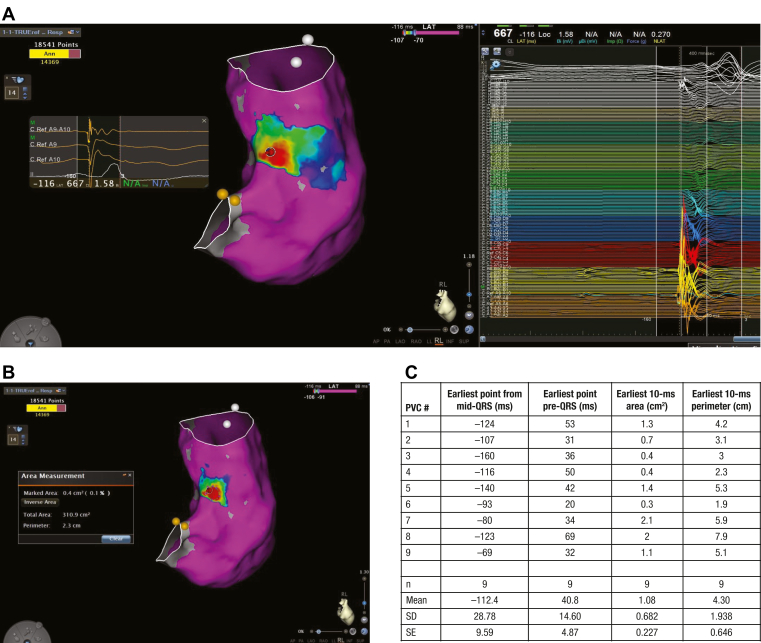
Premature ventricular complex mapping. **A:** Representative PVC map with the earliest QS pre-QRS site was identified at the septal RVOT using the investigational catheter. **B:** The area and the perimeter of the earliest 10-ms points, compared with a map’s reference, were calculated. **C:** A table summarizing all PVC cases identifying the earliest point from mid-QRS (reference), the earliest point pre-QRS, area of the earliest 10-ms points, and perimeter of the earliest 10-ms points. Images are courtesy of © Biosense Webster, Inc, part of Johnson & Johnson MedTech. All rights reserved. PVC = premature ventricular complex; RVOT = right ventricular outflow tract; SD = standard deviation; SE = standard error.

Similarly, points were automatically annotated to the sharpest greatest negative deflection (−dV/dt) and delineating the earliest intracardiac pre-QRS ventricular signal with a QS complex and a sharp downstroke for PVCs. Given that most of the ventricular cases were PVCs and not ischemic VTs, it was not feasible to identify conduction channels, gaps, critical isthmuses, and late potentials. However, the investigational catheter was able to identify and bracket the earliest 10-ms points to an average area of 1.08 cm^2^ for all 9 PVC procedures (Figure 5A–5C).

## Discussion

In recent years, high-density multielectrode mapping catheters have emerged as essential tools for rapidly and accurately delineating arrhythmia mechanisms and enhancing successful clinical outcomes. In this first-in-human feasibility study on 40 patients undergoing catheter mapping and ablation of atrial and ventricular arrhythmias, the investigational ultra-high–density, high-resolution, globe-shaped catheter achieved its primary safety and efficacy endpoints with favorable acute safety and effectiveness profiles for mapping complex arrhythmias. With its deflectable shaft and variable basket deployment, the catheter was able to access all chambers of the heart. For all study procedures, protocol-required preablation mapping was completed with the investigational catheter, with no requirement to switch to another mapping catheter. In this exploratory study on a heterogeneous population, the safety profile of the investigational catheter was comparable with that experienced with other commercially available high-density mapping catheters used for atrial and ventricular procedures. Only 1 SAE within 7 days of the index procedure was deemed related to the investigational catheter; 3 procedure-related SAEs were unrelated to the catheter, whereas 1 nonserious adverse event was considered related to the investigational catheter. The single reported SAE of transient atrioventricular block is likely caused by a mechanical bumping of the compact atrioventricular node, which is relatively common for any catheter when maneuvering adjacent to the atrioventricular node. For all events, participants fully recovered and were discharged within 7 days after the procedure. Median preablation mapping times were 22.4, 22.0, 21.8, 5.4, and 16.8 minutes for participants with AT/AFL, PsAF, PAF, VT, and PVC, respectively.

Like other high-density mapping catheters, the investigational catheter detected PVI breakthroughs and triggers. The investigational catheter design facilitated the rapid identification of mechanisms of the sustaining arrhythmias, which enhanced procedural workflow and efficiency. The presence of 3 embedded magnetic sensors in the distal and proximal portions of the globe that transmit information to the EAM system allowed for basket visualization in an open or closed shape. The catheter’s adaptable size and flat electrodes allowed it to achieve contact in remote areas (eg, ridges, coronary sinus); the catheter should also be atraumatic owing to its architecture (ie, flat-nose tip and flat electrodes). In addition, the location and angle information of the catheter can be transferred to the EAM system independently of the advanced catheter location. Moreover, the combination of an increased number of close-spaced small flat electrodes and an internal noncontact close mapping reference electrode contributes to improved signal quality, offering greater mapping density with a high intracardiac signal resolution. Experience in the current study built on preclinical observations that the close mapping reference electrode was able to filter out far-field ventricular signals during atrial mapping and around lines of block and reduce incorrect timing annotations.^11^^,^^12^ The WofC algorithm was able to reduce outlier misannotated LAT points based on a certain criterion collected within a 1-mm^3^ predefined FAM area (voxel).

Overall, operators rated the investigational catheter as equal to or an improvement over a pentaspline mapping catheter in confirming PVI, its arrhythmogenicity, and tissue characterization. However, the study was limited by having a small sample size of participants spread across different arrhythmia subgroups. Furthermore, interpretation of results, particularly procedural characteristics and operator-rated performance, was limited by the lack of a comparator catheter. Although no direct comparator was included, the operator survey did include questions that asked operators to compare the features of the investigational catheter with their previous experience using a pentaspline mapping catheter and other competitive multielectrode catheters. Participants with the most common arrhythmia mechanisms were expected to enroll in the study rather than those with a range of arrhythmias. Ideally, a greater proportion of patients with reentrant arrhythmias, including ischemic VT cases, could have been added for a fuller assessment of the investigational catheter, and larger studies with a longer follow-up would be a better indication of effectiveness. CARTO version 7 was the latest CARTO version available at the time of this study. Other unique features designed to leverage the investigational catheter’s globe-shaped design were not yet available during the period of the study procedures.

## Conclusion

This novel globe-shaped ultra-high–density, high-resolution mapping catheter successfully mapped complex cardiac arrhythmias and identified key regions of interest. The catheter design and improved software features provided high-resolution signal quality and manual point annotation reduction while maintaining a favorable safety profile. Operator feedback indicated that the catheter successfully met or exceeded physicians’ expectations in terms of deployment, maneuverability, and signal quality acquired for mapping in the atria and ventricles, with physicians particularly appreciating the reduced arrhythmogenicity of this catheter design compared with other devices. Additional studies may demonstrate whether the novel features of the catheter enhance long-term clinical outcomes, but the catheter would not be commercially available.

## Data sharing statement

Johnson & Johnson MedTech has an agreement with the Yale Open Data Access Project to serve as the independent review panel for evaluation of requests for clinical study reports and participant-level data from investigators and physicians for scientific research that will advance medical knowledge and public health. Requests for access to the study data can be submitted through the Yale Open Data Access Project site at http://yoda.yale.edu.

## Disclosures

All authors received support for the present study from Biosense Webster, Inc, part of Johnson & Johnson MedTech. A. Anic received consulting fees and speaking honoraria from Boston Scientific Inc, CardioFocus Inc, and Biosense Webster, Inc; and has contracted research with Boston Scientific Inc, CardioFocus Inc, Biosense Webster, Inc, Arga MedTech, Atacor Medical Inc, Future Cardia Inc, and Bolt Medical Inc. M.G. received payment or honoraria for presentations and has a patent agreement not related to the present study with Biosense Webster, Inc, part of Johnson & Johnson MedTech. T.D.P. received consulting fees and payment or honoraria to his institution from Biosense Webster, Inc, part of Johnson & Johnson MedTech, and served as European Heart Rhythm Association program committee cochair from 2021 to 2022. M.M. received grants as lead investigator for the POLARIS collaborative study; received consulting fees and payment or honoraria for lectures from Biosense Webster, Inc, part of Johnson & Johnson MedTech, Boston Scientific, Abbott, Medtronic, Siemens, Novartis, Janssen, Boehringer Ingelheim, Pfizer, and Sentreheart/Atricure; and has equity in EPD-Philips (divested) and NewPace Ltd. A. Almorad received consulting fees from Biosense Webster, Inc, part of Johnson & Johnson MedTech, Medtronic, Biotronik, and Abbott; received payment or honoraria for lectures from Biosense Webster, Inc, part of Johnson & Johnson MedTech, and Bristol Myers Squibb; and participated on a data safety monitoring board or advisory board for Medtronic and Bristol Myers Squibb.
